# Symptoms, CA125 and HE4 for the preoperative prediction of ovarian malignancy in Brazilian women with ovarian masses

**DOI:** 10.1186/1471-2407-13-423

**Published:** 2013-09-18

**Authors:** Denise da Rocha Pitta, Luis Otávio Sarian, Amilcar Barreta, Elisabete Aparecida Campos, Liliana Lucci de Angelo Andrade, Ana Maria Dias Fachini, Leonardo Martins Campbell, Sophie Derchain

**Affiliations:** 1 Department of Obstetrics and Gynecology, Faculty of Medical Sciences, State University of Campinas – Unicamp, Campinas, SP 13083-970, Brazil; 2 Post Graduating Program in Gynecology, Unicamp, Campinas, Brazil; 3 Department of Pathology, Faculty of Medical Sciences, State University of Campinas – Unicamp, Campinas, SP 13083-970, Brazil

**Keywords:** Specific symptoms, Ovarian tumors, CA125, HE4, ROMA, Prediction of malignancy

## Abstract

**Background:**

This manuscript evaluates whether specific symptoms, a symptom index (SI), CA125 and HE4 can help identify women with malignant tumors in the group of women with adnexal masses previously diagnosed with ultrasound.

**Methods:**

This was a cross-sectional study with data collection between January 2010 and January 2012. We invited 176 women with adnexal masses of suspected ovarian origin, attending the hospital of the Department of Obstetrics and Gynecology of the Unicamp School of Medicine. A control group of 150 healthy women was also enrolled. Symptoms were assessed with a questionnaire tested previously. Women with adnexal masses were interviewed before surgery to avoid recall bias. The Ward Agglomerative Method was used to define symptom clusters. Serum measurements of CA125 and HE4 were made. The Risk of Ovarian Malignancy Algorithm (ROMA) was calculated using standard formulae.

**Results:**

Sixty women had ovarian cancer and 116 benign ovarian tumors. Six symptom clusters were formed and three specific symptoms (back pain, leg swelling and able to feel abdominal mass) did not agglomerate. A symptom index (SI) using clusters abdomen, pain and eating was formed. The sensitivity of the SI in discriminating women with malignant from those with benign ovarian tumors was 78.3%, with a specificity of 60.3%. Positive SI was more frequent in women with malignant than in women with benign tumors (OR 5.5; 95% CI 2.7 to 11.3). Elevated CA125 (OR 11.8; 95% CI 5.6 to 24.6) or HE4 (OR 7.6; 95% CI 3.7 to 15.6) or positive ROMA (OR 9.5; 95% CI 4.4 to 20.3) were found in women with malignant tumors compared with women with benign tumors. The AUC-ROC for CA125 was not different from that for HE4 or ROMA. The best specificity and negative predictive values were obtained using CA125 in women with negative SI.

**Conclusion:**

Women diagnosed with an adnexal mass could benefit from a short enquiry about presence, frequency and onset of six symptoms, and CA125 measurements. Primary care physicians can be thereby assisted in deciding as to whether or not reference the woman to often busy, congested specialized oncology centers.

## Background

Each year, nearly 255.000 new cases of ovarian cancer are diagnosed. Ovarian cancers are the 7th most common type of cancer in women, leading the mortality rate among gynecological cancers by causing 140.000 deaths per year [1]. The incidence of ovarian cancer is higher in industrialized countries, although developing countries, due to larger populations, hold the majority of cases (96.700 *vs* 107.500). In Latin America, the 8/100.000 incidence is close to that of developed countries, which is 10/100.000 women. It was expected that 6.190 ovarian cancer cases would have been diagnosed in Brazil in 2012, with an estimated risk of 6:100.000 women. Not considering non-melanoma skin cancer, ovarian cancer is the seventh most frequent cancer in Brazilian women [2].

In general, ovarian malignancies are diagnosed at an advanced stage, when symptoms are clearly present, or incidentally, at an earlier stage, when an ultrasound is made. It has long been demonstrated that long term survival of ovarian cancer patients is better when these women are treated in specialized training centers, by gynecologists with expertise in gynecologic oncology [3]. In Brazil, a substantial share of the patients is operated by ‘semi-specialized’ gynecologists without formal training but with experience in oncology, generally in high-volume centers specialized in cancer. This professional is likely to be able to perform staging surgery for tumors apparently confined to the ovaries, and debulking surgery for advanced stage disease [3].

The preoperative assessment of an adnexal mass is difficult, leading to a disproportionate number of women with benign ovarian tumors being referred to specialized centers and vice-versa, i.e., women with ovarian cancer being inappropriately operated in non-specialized centers. In a systematic review, Geomini et al. [4] demonstrated that the Risk of Malignancy Indexes (RMI) I and II, which use the product of the serum CA125 level, an ultrasound scan result, and the menopausal state, were the best predictors of malignancy in the preoperative assessment of adnexal masses. Since 1999, the authors of the International Ovarian Tumor Analysis (IOTA) study have been analyzing a large cohort of patients with persistent adnexal masses, in different clinical centers using a standardized ultrasound protocol [5]. Their results consistently showed that using algorithms or even the application of simple and straightforward ultrasound classifications are the most accurate ways of identifying patients with malignant ovarian tumors [5,6]. These algorithms and simple rules have been extensively validated [5,6]. In a recent study we tested the IOTA simple ultrasound rules [7] to identify malignant tumors in women with adnexal masses, resulting in a net sensitivity of 90%, specificity of 87%, positive predictive value (PPV) of 69% and negative predictive value (NPV) of 97% [7]. However, it must be emphasized that the high performance of IOTA-based ultrasound was obtained in the hands of examiners with high level of ultrasound experience. These experienced examiners are more likely to be found in specialized centers.

Recently, many studies examined whether symptoms could help in the selection of women at high risk of harboring a malignant ovarian tumor. More than 90% of women with ovarian cancer report at least one symptom and these symptoms are most often the reason for the visit leading to the diagnosis. However, it remains unknown whether the evaluation of these symptoms is able to discriminate women with malignant ovarian tumors from women with benign adnexal masses [8]. It appears that women with ovarian cancer at any stage are more likely than their counterparts with ovarian benign masses to experience very frequent, sudden onset and persistent symptoms [8-11].

In parallel, CA125 serum measurements may also contribute to the identification of ovarian malignancies, although recent studies suggest that this contribution may be marginal [12,13]. For this reason, novel biomarkers that may help the differentiation of women with malignant tumors are currently under intensive scrutiny [14]. Moore and colleagues [15] have explored a large number of new biomarkers and recently the Food and Drug Administration approved HE4 and the Risk of Malignancy Algorithm (ROMA) for the diagnosis of ovarian cancer in woman with a clinically detectable ovarian mass. However, the diagnostic accuracy of HE4 and ROMA is still controversial. In a recent meta-analysis, Li et al. [14] concluded that although ROMA can help distinguish epithelial ovarian cancer from benign pelvic masses, HE4 is not better than CA125 for ovarian cancer prediction.

In the present study, we investigated whether the preoperative evaluation of specific symptoms and tumor markers in Brazilian women with suspected adnexal masses previously diagnosed with ultrasound may help in the identification of the women who harbor a malignant ovarian tumor. We also evaluated the presence of these symptoms in a group of controls to assess the likelihood of healthy women to experience symptoms associated with adnexal tumors.

## Methods

### Patient selection

This was a cross-sectional study with prospective data collection. The study was approved by the institutional review board of the Unicamp School of Medicine (protocol #1092/2009). An informed consent was obtained from all participants. Women with adnexal masses of suspected ovarian origin attending the hospital of the Department of Obstetrics and Gynecology of the Unicamp School of Medicine were invited to enroll. A control group of healthy women attending menopause and family planning clinics at the same hospital was selected. As soon as surgery was indicated, women who had adnexal masses received an explanation about the study methods and purpose. Symptoms were assessed with a questionnaire previously tested and published by Goff et al. [9]. The questionnaire was applied to all women, in-person, by a trained professional (DRP). Women with adnexal masses were interviewed before surgery to avoid recall bias, since the main purpose of the study was to investigate whether symptoms could help to preoperatively discriminate women with malignant ovarian tumors. We also collected data on age and body mass index (BMI). Peripheral blood was collected for serum measurements of CA125 and HE4. The mean time elapsed from interview, blood collection to surgery ranged 24 h or less for emergency procedures to a maximum of 120 days. Exclusion criteria comprised women who had already been operated for the adnexal mass and ongoing pregnancy. The final sample of this study consisted of 176 women with adnexal masses of ovarian origin and 150 healthy women. Patient accrual ranged January 2010 – January 2012, and collection of data regarding the marker status and pathological diagnoses lasted through May 2012.

### Symptoms

As previously stated, women with adnexal masses were surveyed prior to surgery, before they knew their histological diagnosis. The survey evaluated the presence, frequency and duration of pelvic pain, abdominal pain, back pain, indigestion, being unable to eat normally, feeling full quickly, having nausea or vomiting, weight loss, abdominal bloating, increased abdomen size, being able to feel abdominal mass, urinary urgency, frequent urination, constipation, diarrhea, menstrual irregularity, bleeding after menopause, pain during intercourse, bleeding with intercourse, fatigue, leg swelling, and difficulty breathing. The survey was originally designed in English and was submitted to a Portuguese translation, which included two forward translations, one reconciled version and a back translation of the reconciled version. Initially, the patient was questioned about the presence or absence of a symptom. If present, the severity of each symptom along with its frequency and duration were evaluated. The frequency was reported with respect to the number of days per month, classified as: <1, 1–2, 3–6, 7–12, 13–19 or >20 days/month. The duration was reported with respect to how long the symptom persisted. Next, the patient was asked during how many of the previous 12 months did the symptom occur, which was further categorized in <1, 1–2, 3–4, 5–6, 7–9, 10–12, >12 months. This symptom categorization emphasizes onset and frequency, since previous studies demonstrated that these two features are strongly related to malignancy [9,11]. We considered a symptom positive if it occurred more than 12 times per month, beginning in the last year, regardless of this severity [9,16].

### Serum samples and marker assays

Blood samples were collected from all patients and stored in Serum Separator Tubes (SST). They were allowed to clot for at least 30 minutes before centrifugation. The blood samples were centrifuged 1300 g for 10 min, and serum was aliquoted and stored at −80°C until analysis. Automated analysis of CA125 was performed by solid phase chemiluminescence using the OM-MA test (Siemens Medical Solutions Diagnostics, Tarrytown, USA) according to the manufacturer’s instructions and using their reagents and equipment. Values were expressed in units per milliliter (U/mL). We used the Immunochemiluminometric assay ([ICMA], Immulite® 2000 OM-MA, Siemens Medical Solutions Diagnostics) for CA125 measurements. The ROMA™ preconizes the use of the ARCHITECT CA125 II™ assay, which is a Chemiluminescent Microparticle Immunoassay (CMIA), essentially the same technology as ICMA. According to Li et al. [14] CA125 tests with EIA (enzyme immunoassay) and RIA (radioimmunoassay) are considered “High Concern Regarding Applicability”. CMIA and ICMA are thus equivalent technologies that can be used interchangeably. The level of serum HE4 was determined using the HE4 enzyme immunometric assay Kits (EIA) (Fujirebio Diagnostics, Göteborg, Sweden) based on the direct sandwich technique, solid-phase immunoassay according to the manufacturer’s instructions and using their reagents and equipment. Values were expressed in picomoles per liter (pMol/L).

#### Calculation of the Risk of Ovarian Malignancy Algorithm (ROMA)

The Risk of Ovarian Malignancy Algorithm (ROMA™) uses the ABBOT ARCHITECT™ platform results for HE4 and CA125 to generate a predictive index (PI) for epithelial ovarian cancer, calculated by the formulae proposed by Moore et al. [15] for pre-menopausal and post-menopausal women. The manufacturer recommends the ROMA™ index to be used to stratify women into high-risk or low-risk groups of having epithelial ovarian cancer (EOC). We decided to use ROMA for the discrimination of women with ovarian malignancies, not only EOC. The ROMA™ risk estimation is based on the ABBOT ARCHITECT™ platform; however, since we used the OM-MA test for CA125 (Siemens Medical Solutions Diagnostics, Tarrytown, USA) and the HE4 EIA Kit (Fujirebio Diagnostics), differences in assay methods and reagent specificity could lead to different performances. Thus, we decided to use cutoff points based on the essay performance obtained with our sample (see statistics).

#### Surgery and pathological assessment of tumor specimens

Surgeries for diagnosis and/or treatment were performed at the hospital of the Department of Obstetrics and Gynecology of Unicamp School of Medicine and the techniques and surgical procedures were chosen and performed according to medical indication. All women with ovarian cancer were fully staged. The gold standard was the histopathologic diagnosis of surgical specimens, rendered by pathologists of the Department of Pathologic Anatomy of the Unicamp School of Medicine, following the guidelines of the World Health Organization International Classification of Ovarian Tumors [17]. For statistical purposes, the epithelial borderline tumors were classified as malignant (i.e. 10 out of 47 epithelial malignant tumors were rendered as borderline).

#### Statistical analysis

Data were entered into a Microsoft Excel (Microsoft Corp., Redmond, WA, USA) spreadsheet and analyzed with the R Environment for Statistical Computing Software® [18]. All statistical calculations were performed using 95% confidence intervals (CIs) and *P* <0.05 was considered significant. Women were classified into benign and malignant groups according to tumor histologic diagnoses. The sample size was calculated on the basis of the difference in symptom prevalence derived from previous studies [19,20], with 5% significance levels, 80% statistical power and 12% error limits for the sensitivity. Using these parameters, the minimal number of women with malignant tumors would be 54, and based on the prevalence of malignancy, 112 women with benign tumors would be needed for discrimination.

#### Data analysis plan

We first compared the main clinical features of the women in the three study groups using chi-squares, and the Kruskal-Wallis test for continuous numerical variables such as age and BMI. Pairwise comparisons were done: women with malignant tumors vs. those with benign tumors; malignant vs. controls, and benign vs. controls. Next, using the pairwise groupings listed before, we compared the proportions of women presenting with each of the 22 specific symptoms. A dichotomous classification for each symptom was used: positive if the symptoms had occurred more than 12 times, beginning in the last year, regardless of its severity; negative if otherwise. The proportions were pairwise compared using chi-squares or the Fisher exact test where appropriate. Because the prevalence of symptoms was very low in control women, this group was excluded from the subsequent analyses (Table 1).

**Table 1:** Specific symptoms in women with malignant or benign ovarian tumors and controls (healthy women)

	**Malignant**		**Benign**		**Controls**		**p-value**	**p-value**	**p-value**
	**tumors**		**tumors**						
	**(n = 60)**		**(n = 116)**		**(150)**		**(Malignant vs. benign)**	**(Malignant vs. controls)**	**(Benign vs. controls)**
**Symptom**	**N**	**(%)**	**n**	**(%)**	**n**	**(%)**			
Pelvic pain	30	(50.0)	23	(19.7)	2	(1.3)	<0.01	<0.01	<0.01
Abdominal pain	23	(38.3)	10	(8.5)	0	-	<0.01	<0.01	<0.01
Back pain	17	(28.3)	10	(8.6)	0	-	<0.01	<0.01	<0.01
Unable to eat normally	22	(36.7)	8	(6.8)	1	(0.7)	<0.01	<0.01	0.01
Feeling full quickly	22	(36.1)	11	(9.4)	1	(0.7)	<0.01	<0.01	<0.01
Indigestion	15	(24.6)	7	(6.0)	1	(0.7)	<0.01	<0.01	0.02
Nausea or vomiting	11	(18.0)	4	(3.4)	1	(0.7)	<0.01	<0.01	0.17
Weight loss	19	(31.7)	25	(21.6)	11	(7.3)	0.19	<0.01	<0.01
Abdominal bloating	36	(60.0)	32	(27.4)	0	-	<0.01	<0.01	<0.01
Increased abdomen size	38	(63.3)	31	(26.7)	1	(0.7)	<0.01	<0.01	<0.01
Able to feel abdominal mass	14	(23.3)	11	(9.4)	0	-	0.02	<0.01	<0.01
Urinary urgency	8	(13.3)	8	(6.8)	2	(1.3)	0.25	<0.01	0.02
Frequent urination	12	(20.0)	16	(13.7)	2	(1.3)	0.38	<0.01	<0.01
Constipation	2	(3.3)	2	(1.7)	0	-	0.60	0.08	0.18
Diarrhea	3	(5.1)	1	(0.9)	0	-	0.11	0.02	0.43
Menstrual irregularities*	1	(1.6)	1	(0.9)	1	(0.7)	1.0	0.49	1.0
Bleeding after menopause**	6	(10.0)	1	(0.9)	0	-	<0.01	<0.01	0.43
Pain during intercourse***	0	-	0	-	1	(0.7)	NC	1.0	1.0
Bleeding with intercourse	0	-	0	-	0	-	NC	NC	NC
Fatigue	20	(33.3)	16	(13.7)	3	(2.0)	<0.01	<0.01	<0.01
Leg swelling	6	(10.0)	11	(9.4)	1	(0.7)	1.0	<0.01	<0.01
Difficulty breathing	9	(15.0)	5	(4.3)	2	(1.3)	0.02	<0.01	0.24

*only for premenopausal women; **only for postmenopausal women; ***only for sexually active women; *NC* non-computable.

P-value: bivariate pairwise comparisons using chi-squares or the Fisher’s exact test where appropriate.

#### Determination of symptom clusters

**Figure 1:**
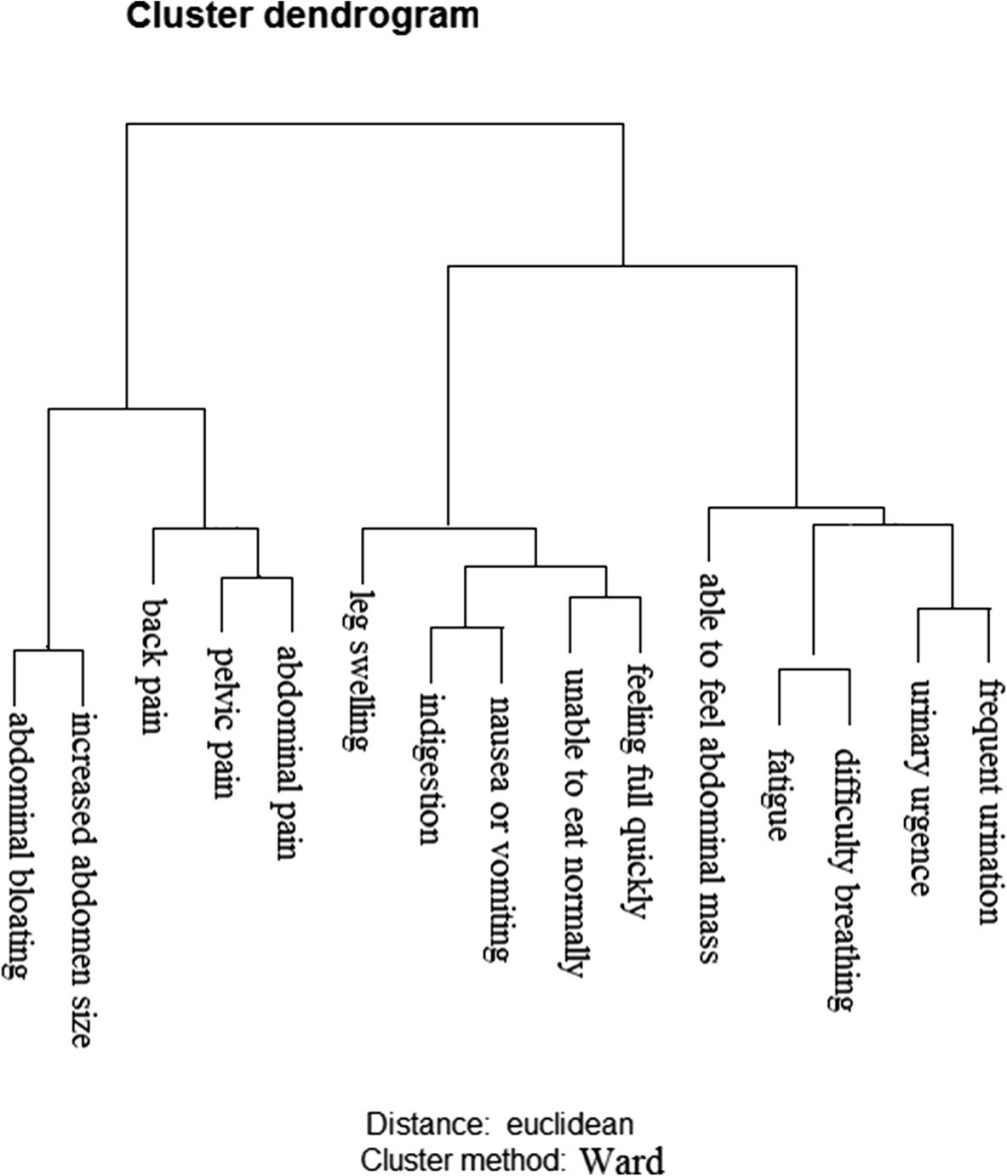
**Ward agglomerative method for hierarchical clustering.** The following clusters of symptoms and isolated symptoms were defined by the Ward agglomerative method: abdomen (abdominal bloating and/or increased abdominal size); back pain; pain (pelvic and/or abdominal pain); leg swelling; eating (unable to eat normally and/or feeling full quickly); able to feel abdominal mass; miscellaneous (fatigue and/or difficulty breathing); digestion (indigestion and/or nauseas/vomiting); bladder (urinary urgency and/or frequent urination).

The Ward’s Hierarchical Clustering Method [21] was used to evaluate whether the specific symptoms could be clustered in women with malignant or benign ovarian tumors. The following specific symptoms were not included in the Ward’s model: menstrual irregularity, bleeding after menopause, pain during intercourse, and bleeding with intercourse, because these symptoms depend on menopausal status and sexual activity; constipation and diarrhea, because these symptoms appeared very rarely; and weight loss, because frequency could not be ascertained for that symptom. Thus, sixteen specific symptoms were entered into the Ward model. This method allows for the formation of statistically significant agglomerates of symptoms, which were depicted in the Euclidian plane (Figure 1): related symptoms appear close to each other; the closer they are, the more related to each other. We compared the prevalence of the symptom clusters and the remaining isolated symptoms in women with either malignant or benign tumors using crude (unadjusted) odds ratios and chi-squares/Fisher Exact test. We also calculated the performance indicators (sensitivity, specificity, with 95% confidence intervals, positive and negative predictive values – PPV and NPV) for each symptom cluster and isolated symptom in discriminating malignant from benign tumors. Goff et al. (2007) [9] proposed a “symptom index” (SI) that was most predictive of a women having ovarian cancer; the SI is considered positive if the women has at least one of the following symptom groupings: abdominal or pelvic pain, feeling full quickly or unable to eat normally, or increased abdomen size. Coincidentally, in our study, these symptoms formed identical clusters and were the most sensitive and prevalent. We thus decided to replicate Goff’s SI in our study.

#### Determination of CA125, HE4 and the ROMA predictive index cutoff points

We used standard receiver operator characteristics (ROC) analysis to determine the best CA125, HE4 and ROMA index cutoff points in discriminating benign from malignant ovarian tumors. In premenopausal women, the optimal cutoff points for CA125, HE4 and ROMA predictive index were, respectively, 69.8 U/L, 41.6 pmol/L and 5.01%. In postmenopausal women these cutoff points were, respectively, 21.7 U/L, 96.6 pmol/L and 18.2%. ROC AUC comparisons were performed with the DeLong method [22].

#### Accuracy of symptom clusters, symptom index and tumor markers

We performed pairwise comparisons of prevalence of the symptom clusters, symptom index, and the positivity rate of CA125, HE4 and ROMA index according to tumor malignancy and stage strata, using unadjusted odds ratios with 95% CI. Next, we calculated the performance indicators (sensitivity, specificity, with 95% confidence intervals, positive and negative predictive values) for the symptom clusters and tumor markers using standard formulae.

## Results

Table 2 shows the comparison of key clinical features of women with malignant or benign ovarian tumors and controls. The mean age was significantly higher in women with malignant tumors. BMI was balanced between the study groups. Epithelial benign and malignant tumors prevailed over the other histological types, but germ line (mature teratomas) and stromal tumors (fibromas) were also common in women with benign tumors. More than 50% of the women with malignant tumors had stage I disease.

**Table 2:** Key clinical features of women with ovarian malignant tumors, ovarian benign tumors and healthy women (controls)

**Characteristics**	**Malignant**	**Benign**	**Controls**	**p-value**	**p-value**	**p-value**
	**(n = 60)**	**(n = 116)**	**(n = 150)**	**(Malignant vs. benign)**	**(Malignant vs. controls)**	**(Benign vs. controls)**
*Age*						
Years, mean (SD)*	50.8 (20.5)	47.9 (16.6)	44.4 (12.6)	0.45	0.02	0.18
*Menopausal status*^*Ŧ*^						
Premenopausal	23 (38.3)	68 (57.8)	79 (52.6)			
Postmenopausal	38 (61.7)	49 (42.2)	71 (48.4)	<0.01	0.05	0.37
*Body mass index (BMI) – kg/m*^*2*^						
Mean (+/− SD)*	28.3 (+/−6.3)	28.0 (+/−5.5)	27.2 (+/−5.0)	0.42	0.37	0.40
*Histology*						
Epithelial	47 (78.3)	40 (34.5)	-	-	-	-
*Invasive*	*37 (78.7)*					
*Borderline*	*10 (21.3)*					
Germ line	6 (10.0)	28 (24.1)	-	-	-	-
Sex cord and Stromal	7 (12.7)	20 (17.3)	-	-	-	-
Non-neoplasic	-	28 (24.1)	-	-	-	-
*Stage*						
I	32 (53.3)					
II	5 (8.3)					
III	22 (36.7)					
IV	1 (1.7)					

p values calculated with either chi-squares^*Ŧ*^ or the Kruskal-Wallis test*.

*2 ovarian carcinomas had endometrial carcinoma associated; for these cases ovarian carcinoma was considered the primary tumor.

Women with malignant tumors showed a higher frequency of symptoms such as pelvic pain, abdominal pain, back pain, being unable to eat normally, feeling full quickly, indigestion, abdominal bloating, increased abdominal size, being able to feel abdominal mass and fatigue when compared with women with benign ovarian tumors. The prevalence of symptoms in control women was very low, with the exception of weight loss. This fact led us to exclude controls from the subsequent analyses (Table 1).

Figure 1 shows the Euclidian representation of the Ward Agglomerative Method used to define the symptom clusters. This method was able to define 6 different clusters of symptoms. These clusters were named as follows: abdomen (agglomeration of the following specific symptoms: abdominal bloating and/or increased abdominal size); pain (pelvic and/or abdominal pain); digestion (indigestion and/or nauseas/vomiting); eating (unable to eat normally and/or feeling full quickly); miscellaneous (fatigue and/or difficulty breathing) and bladder (urinary urgency and/or frequent urination). Three specific symptoms (back pain, leg swelling and able to feel abdominal mass) did not agglomerate and remained as isolated symptoms.

Table 3 compares the prevalence of symptom clusters and isolated symptoms in women with benign or malignant ovarian tumors. Clusters and isolated symptoms were sorted according to decreasing prevalence in the studied population. With the exception of the cluster bladder and the isolated symptoms leg swelling, all symptoms were significantly more prevalent in women with malignant tumors.

**Table 3:** Prevalence of symptoms clusters/isolated symptoms according to tumor malignancy

**Symptoms**	**Malignant positive N (%)**	**Benign positive N (%)**	**Crude odds ratio (95% CI)**	**p**
Cluster abdomen	41(68.3)	36 (31.0)	4.7 (2.4 to 9.3)	<0.01
Cluster pain	33 (55.0)	26 (22.4)	4.2 (2.1 to 8.2)	<0.01
Cluster eating	27 (45.0)	14 (12.0)	5.9 (2.8 to 12.6)	<0.01
Cluster miscellaneous	21 (35.0)	18 (15.4)	2.9 (1.4 to 6.0)	0.01
Cluster digestion	18 (30.0)	10 (8.5)	4.5 (1.9 to 10.5)	0.01
Back Pain	17 (28.3)	10 (8.6)	4.1 (1.8 to 9.8)	<0.01
Able to feel abdominal mass	14 (23.3)	11 (9.4)	2.9 (1.2 to 6.8)	0.02
Cluster bladder	14 (23.3)	20 (17.1)	1.4 (0.7 to 3.1)	0.42
Leg swelling	6 (10.0)	11 (9.4)	1.1 (0.4 to 3.0)	1.0

Clusters of symptoms and isolated symptoms were defined by the Ward agglomerative method: abdomen (abdominal bloating and/or increased abdominal size*)*; pain *(*pelvic and/or abdominal pain); eating (unable to eat normally and/or feeling full quickly); miscellaneous (fatigue and/or difficulty breathing); digestion (indigestion and/or nauseas/vomiting); back pain; able to feel abdominal mass; bladder (urinary urgency and/or frequent urination), leg swelling.

Table 4 compares the performance of the symptom clusters, isolated symptoms, and SI in discriminating women with malignant ovarian tumors from the others. Clusters and symptoms were ordered from the most to the least sensitive. Clusters abdomen, pain and eating were the most sensitive and those with the best PPV, and were therefore chosen to be used in the symptom index (SI) calculation. The sensitivity of the SI in discriminating women with malignant from those with benign ovarian tumors was 78.3%, with a specificity of 60.3%.

**Table 4:** Performance of cluster of symptoms and the symptom index for the differentiation of women with malignant ovarian tumors from women with benign tumors

**Symptoms**	**Sensitivity (%) (95% CI)**	**Specificity (%) (95%)**	**PPV (%)**	**NPV(%)**
Cluster abdomen	68.3 (59.9 to 63.1)	69.0 (58.6 to 79.3)	80.8	53.2
Cluster pain	55.0 (45.0 to 64.0)	77.8 (67.2 to 88.4)	77.1	55.0
Cluster eating	45.0 (36.0 to 54.0)	88.0 (78.1 to 98.0)	75.7	65.8
Cluster miscellaneous	35.0 (26.3 to 43.6)	84.6 (73.3 to 96.0)	71.7	53.8
Cluster digestion	29.5 (21.2 to 37.8)	91.4 (81.1 to 100.0)	71.3	64.3
Back Pain	28.3 (20.1 to 36.5)	91.4 (80.8 to 100)	71.1	63.0
Able to feel abdominal mass	23.3 (15.7 to 31.0)	90.6 (79.1 to 100)	69.7	56.0
Cluster bladder	23.3 (15.7 to 31.0)	82.9 (70.2 to 95.6)	67.8	41.2
Leg swelling	10.0 (4.6 to 15.4)	90.6 (76.7 to 100)	66.2	35.3
Symptom index (SI)	78.3 (70.8 to 85.8)	60.3 (5.4 to 70.3)	50.5	84.3

Clusters of symptoms and isolated symptoms were defined by the Ward agglomerative method: abdomen (abdominal bloating and/or increased abdominal size); pain (pelvic and/or abdominal pain); eating (unable to eat normally and/or feeling full quickly); miscellaneous (fatigue and/or difficulty breathing); digestion (indigestion and/or nauseas/vomiting); back pain; able to feel abdominal mass; bladder (urinary urgency and/or frequent urination), leg swelling. Symptom index (SI) = presence of at least one of the symptoms included in the clusters abdomen, pain and/or eating.

In Table 5, we compared the prevalence of the three most sensitive symptom clusters, the SI, and the positivity rate of CA125, HE4, and ROMA predictive index across histological and stage strata. The percentage of women with ovarian malignancy who experienced at least one cluster of symptoms ranged 37% to 72%, and this prevalence was not significantly associated with disease stage. The proportion of women with positive SI did not vary significantly across disease stage strata, with figures around 78%. The proportion of women with positive SI was also significantly lower in women with benign tumors compared to women with stage I disease. Women with malignant tumors had significantly more elevated levels of the tumor markers compared to women with benign tumors. However, only 34% of the women with stage I disease had positive ROMA predictive index (PI), contrasted to 84% in women with advanced stage disease. It is worth noting, 40% of the women with benign tumors had positive SI, but only 12% of these women had positive ROMA PI.

**Table 5:** Prevalence of symptoms and tumor markers as related to tumor malignancy and stage

	**Malignant**			**Benign**	**OR (95% CI)**			
	**Stage I**	**Stage II-IV**	**All**	**(n = 116)**	**All malignant**	**Stage I**	**Stage II-IV**	**Stage II-IV**
	**(n = 32)**	**(n = 28)**	**(n = 60)**		**vs. benign**	**vs. benign**	**vs. benign**	**vs. stage I**
Cluster	%	%	%	%				
Abdomen	72	64	68	31	4.7 (2.4 to 9.4)	5.7 (2.4 to 13.5)	4.0 (1.7 to 9.5)	0.7 (0.2 to 2.1)
Pain	44	68	55	22	4.2 (2.1 to 8.2)	2.7 (1.6 to 6.1)	7.3 (2.9 to 18.1)	2.7 (0.9 to 7.8)
Eating	37	54	45	12	6.0 (2.8 to 12.7)	4.4 (1.8 to 10.8)	8.4 (3.3 to 21.3)	1.9 (0.7 to 5.4)
Symptom index (SI)	78	79	78	40	5.5 (2.7 to 11.3)	5.4 (2.2 to 13.6)	5.6 (2.1 to 14.8)	1.0 (0.3 to 3.5)
CA125	47	85	65	24	5.7 (2.8 to 12.2)	3.5 (1.5 to 9.2)	18.4 (5.4 to 79.4)	5.1 (1.3 to 25.1)
HE4	34	86	58	15	7.6 (3.7 to 15.6)	2.8 (1.2 to 6.9)	32.7 (10.1 to 105.4)	11.4 (3.2 to 41.4)
ROMA PI	34	82	57	12	9.5 (4.4 to 20.3)	3.8 (1.5 to 9.6)	33.5 (11.0 to 102.4)	8.7 (2.6 to 29.5)

Symptom index (SI) = presence of at least one of the symptoms included in the clusters abdomen, pain and/or eating.

Cutoff points in premenopausal women: CA125 = 69.8 U/ml, HE4 = 41.6 pMol/L, ROMA predictive index (PI) = > 5.01%. In postmenopausal women: CA125 = 21.7 U/L, HE4 = 96.6 pMol/L, ROMA-PI > 18.2%. *PPV* positive predictive value, *NPV* negative predictive value.

In Table 6 we evaluated the performance of the tumor markers in differentiating women with malignant tumors (or only women with stage I disease) from women with benign tumors in subsets of women with different symptom patterns. The AUC-ROC for CA125 was not significantly different from that for HE4 or ROMA in discriminating malignant (all stages) or only stage I tumors from benign tumors. The tumor markers yielded their best NPV and specificity in women with negative SI. Using the tumor markers in addition to the SI (the stand-alone performance of the SI is shown in Table 4) increases the specificity and the PPV of the differentiation strategy for malignant (all stages) from benign ovarian masses, but this does not hold true if we want to differentiate stage I disease from benign tumors.

**Table 6:** Performance comparison of tumor markers in subsets of women with adnexal tumors (benign or malignant) with different symptom patterns

**Group**	**Marker**	**ROC - AUC**	**ROC-AUC comparison**			**Sensitivity (%)**	**Specificity (%)**	**PPV (%)**	**NPV(%)**
			**HE4 vs. CA125**	**HE4 vs. ROMA**	**CA125 vs. ROMA**	**(95% CI)**	**(95%)**		
**For the differentiation between all malignant tumors from benign tumors (healthy women not included)**									
All women	CA 125	0.81 (0.75 to 0.89)				65.0 (56.3 to 73.7)	75.9 (65.6 to 86.1)	58.2	80.7
	HE4	0.74 (0.66 to 0.82)	0.09	0.06	0.37	58.3 (49.4 to 67.3)	84.5 (74.7 to 94.2)	66.0	79.7
	ROMA	0.78 (0.71 to 0.86)				56.7 (47.6 to 65.7)	87.9 (78.7 to 97.1)	70.8	79.7
SI Negative	CA 125	0.69 (0.52 to 0.86)				46.1 (34.5 to 57.8)	87.1 (70.2 to 100)	40.0	89.7
	HE4	0.69 (0.54 to 0.84)	0.98	0.45	0.67	53.1 (42.2 to 65.5)	84.3 (67.5 to 100)	38.9	90.1
	ROMA	0.72 (0.57 to 0.87)				38.5 (27.1 to 49.8)	91.4 (74.9 to 100)	45.0	90.7
SI Positive	CA 125	0.81 (0.73 to 0.90)				70.2 (57.0 to 83.4)	58.7 (45.3 to 72.1)	63.5	65.8
	HE4	0.75 (0.65 to 0.85)	0.27	0.25	0.52	59.6 (45.4 to 73.8)	84.8 (72.9 to 96.7)	80.0	67.2
	ROMA	0.78 (0.69 to 0.88)				61.7 (47.6 to 75.7)	82.6 (70.4 to 94.2)	78.4	67.9
**For the differentiation between women with Stage I cancer from those with benign tumors**									
All women	CA 125	0.73 (0.63 to 0.83)				46.8 (37.8 to 56.0)	75.9 (63.1 to 88.7)	34.9	83.8
	HE4	0.61 (0.50 to 0.71)	0.06	0.09	0.25	34.4 (25.7 to 43.0)	84.5 (71.3 to 97.7)	37.9	82.3
	ROMA	0.66 (0.55 to 0.74)				34.4 (25.7 to 43.0)	87.9 (75.2 to 100)	44.0	82.9
SI Negative	CA 125	0.52 (0.32 to 0.73)				14.3 (6.1 to 22.5)	87.1 (66.4 to 100)	10.0	91.0
	HE4	0.60 (0.39 to 0.81)	0.67	0.90	0.66	28.6 (18.0 to 39.1)	84.3 (69.5 to 100)	15.4	92.2
	ROMA	0.61 (0.40 to 0.81)				14.3 (6.1 to 22.5)	91.4 (70.9 to 100)	14.3	91.4
SI Positive	CA 125	0.75 (0.63 to 0.86)				56.0 (41.7 to 70.3)	58.7 (41.9 to 75.5)	42.4	71.0
	HE4	0.58 (0.44 to 0.74)	0.07	0.20	0.22	36.0 (22.1 to 49.9)	84.8 (67.2 to 100)	56.2	70.9
	ROMA	0.65 (0.50 to 0.80)				40.0 (25.8 to 54.2)	82.6 (65.1 to 100)	55.5	71.7

Symptom index (SI) positive = presence of at least one of the symptoms included in the clusters abdomen, pain and/or eating. Cutoff points for each tumor marker were obtained with ROC analysis. Cutoff points in premenopausal women: CA125 = 69.8 U/ml, HE4 = 41.6 pMol/L, ROMA predictive index (PI) = > 5.01%. In postmenopausal women: CA125 = 21.7 U/L, HE4 = 96.6 pMol/L, ROMA-PI > 18.2%. *PPV* positive predictive value, *NPV* negative predictive value.

## Discussion

In this sample of Brazilian women who underwent surgery due to a suspected adnexal mass, the evaluation of specific symptoms proved to be a powerful tool for the discrimination of malignant from benign ovarian tumors. The addition of CA125 to the SI increased the specificity and predictive values for the discrimination of malignant from benign ovarian tumors. This is especially important in a country where most women with adnexal masses have their condition detected with ultrasound in primary health care facilities. Symptom investigation, followed by CA125 serum level assessment is an affordable and straightforward approach to the initial triaging of women at elevated risk of harboring ovarian cancer. This approach can yield a 63% probability that women referred to specialized centers (i.e., if one refers women with positive SI and elevated CA125) indeed have an ovarian malignancy. On the other hand, 90% of the women with an adnexal mass, negative SI and negative CA125 levels will ultimately be found to have a benign ovarian tumor.

Our methodology to evaluate symptom cluster formation yielded results that closely match Goff et al. recent results [11]. As they suggested, we can restrict the symptom questionnaire to a shortened version of six questions encompassing the specific symptoms bloating, increased abdomen size, feeling full quickly, unable to eat normally and abdominal/pelvic pain. It is worth mentioning, however, that diagnostic models are known to deliver good results in the population at which they are first developed. But it must be emphasized that, replicating the methodology and using the same instrument that Goff et al. [9] used in their seminal studies, we obtained similar performance indicators for isolated symptoms and symptom clusters. Using the SI, Goff et al. [11] obtained an overall sensitivity and specificity of 70% and 86%, respectively, for the discrimination of women with ovarian cancer from healthy controls. We, on the other hand, used the SI in women already diagnosed with an adnexal mass, and aimed at discriminating those with a malignancy from the rest. In this context, we obtained a sensitivity of 78% and a specificity of 60%. In our study, the overall prevalence of cancer was 34%, which implies that with a sensitivity of 78% using the SI as a standalone diagnostic tool, approximately 50% of the women referred to a specialized center will ultimately have cancer. On the other hand, only 15% of the women not referred will have cancer. By adding CA125 to the strategy, we may improve the positive predictive value and further reduce the number of women erroneously referred to a specialized center, even if we want to refer women with early stage disease (see Tables 4 and 6).

In the last decade, many studies addressed the symptom experience of women with ovarian cancer, and ovarian cancer can no longer be considered a disease that does not produce symptoms [9,16,23,24]. Women with ovarian cancer may experience various symptoms; however, many of these symptoms have no relationship with the genital tract. Because these symptoms are unspecific, women and physicians tend to underestimate their importance. Women are often treated for irritable bowel syndrome, stress, depression or gastritis, months before they are diagnosed with ovarian cancer [11]. The underrating of symptoms by women and doctors may contribute to ovarian cancer not being timely referred to specialized centers. In our sample, all women with malignancies reported some sort of symptom, which is consistent with data from other populations.

The role to be played by HE4 and ROMA and their significance regarding changes in medical practice are still under debate [14,25]. Andersen et al. [16] in a prospective study comparing 74 women with ovarian cancer and 137 healthy women found out that either CA125 or HE4, when combined with the SI, detected 91.9% of the cases of malignancy. It is now clear that HE4 is essentially useful to distinguish epithelial ovarian cancer from other malignant ovarian tumors. Neither stromal nor germ cell tumors express HE4 and thereby are not distinguishable from benign tumors by using HE4 [14,25]. We analyzed HE4 and ROMA considering all histologic types, because our objective was to identify women that would benefit from a referral to a specialized cancer center. Our conclusion was that HE4 and ROMA did not facilitate the discrimination of malignant from benign ovarian tumors further than CA125 alone.

Our data demonstrated that symptoms may be used even to differentiate women with early stage ovarian cancer from those with benign ovarian tumors. In the present study, 53% of the patients had stage I disease, regardless of the histological type of the tumor, and 78% of these had positive SI. Rossing et al. [26] demonstrated that the SI was positive in 62.3% of women with early stage disease and Goff et al. [27] obtained 57% sensitivity using the SI. However, in both studies, women were surveyed after diagnosis, whereas in our study we surveyed the women before surgery and thus before they were informed of the diagnosis of cancer. Because we aimed at identifying women who would benefit from a referral to a specialized center, we grouped together women with epithelial, germ cell and sex cord malignant tumors. On the other hand, we allocated to a same group women with borderline epithelial tumors and those with low- or high-grade invasive epithelial carcinomas. It is well known that these different histological types display varying clinical behaviors. Based on a dualistic model of carcinogenesis, epithelial ovarian carcinoma can be classified as type I and type II. Type I included low-grade epithelial carcinomas, generally indolent and easily detected in stage I. Type II ovarian carcinoma, comprise high-grade and undifferentiated carcinomas [28]. It has been well demonstrated that high-grade serous tumors are rarely diagnosed before they had spread, and for this type of tumors, diagnostic approaches should be aimed at diagnosing low-volume tumors, not only tumors at an early stage [29,30].

### Strengths and limitations

The main strengths of this study are that we made all the interviews and tumor marker collection before surgery avoiding recall bias. All participants were interviewed in person with a standardized questionnaire. We also took care to assess symptoms in a relatively large cohort of healthy women that had attended family planning and menopause-related medical consultations at the same center. We found that these women have a very small likelihood of experiencing symptoms of recent onset and high frequency, which led us to safely remove these women from symptom performance calculations. We must also mention that the questionnaire for the characterization of symptoms was used in Latin American women for the first time, and the results encountered match those from studies addressing women from different cultural backgrounds [8,10,11].

As a detrimental point, our study suffers from verification bias, since we needed the final pathological diagnoses for analyses and therefore only women who were operated for their adnexal masses had been evaluated. The performance of symptoms and serum markers was not evaluated in women who were not operated. Another limitation of our study resides in the fact that we have not analyzed pre and postmenopausal women separately. Of course, in our analyses, symptoms which applied only to pre- or postmenopausal women and those applicable only to sexually active women were not included in the multivariate models. Unfortunately, this approach is not sufficient to rule out this selection bias because, for example, pelvic pain, which was significantly associated with malignancy, is frequently reported by young women with endometrioma [31,32]. As another weakness of the study, HE4 levels are known to be associated with BMI and age, but our analyses did not control for these variables.

## Conclusion

Neither symptoms nor CA125 can be safely used as standalone instruments to discriminate women with malignant ovarian tumors from women with benign adnexal masses in lieu of a well performed ultrasound examination of the pelvis. However, in the foreseeable future, it is not realistic to expect that such a well performed ultrasound would be widely available in primary care facilities, even if we consider that IOTA simple rules can substantially increase overall ultrasound performance, at the same time simplifying sonographer training [5,7]. Collectively, our data indicate that asking a woman, who already had an adnexal mass incidentally detected in ultrasound, about the presence, frequency and onset of six symptoms and determining CA125 levels can facilitate the decision making of primary care physicians as to whether or not reference the women to often busy, congested specialized oncology centers.

## Competing interests

The authors declare that they have no competing interests.

## Authors’ contributions

DRP and EAC conducted the experiments and prepared the manuscript, under the supervision of SD and LOS, who also designed the study. EAC gave technical advice, AB and AMDF contributed with the acquisition of data and also provided clinical advice during manuscript preparation. LLAA contributed on pathological advice. LMC revised the final text. All authors read and approved the final manuscript.

## Pre-publication history

The pre-publication history for this paper can be accessed here:

http://www.biomedcentral.com/1471-2407/13/423/prepub
